# Attenuation of Influenza a Virus into Live Vaccines Through C‐End Degrons

**DOI:** 10.1002/advs.202509425

**Published:** 2026-02-21

**Authors:** Ping Wang, Le Li, Yunfang Chen, Le Tong, Zhen Li, Rong Yu, Quan Shen, Qikai Wang, Jihuan Hou, Qisi Zhang, Xu Si, Ning Wang, Demin Zhou, Wen‐xia Tian, Longlong Si

**Affiliations:** ^1^ Shanxi Key Laboratory of Animal Disease Research, Prevention and Control, College of Veterinary Medicine Shanxi Agricultural University Jinzhong China; ^2^ State Key Laboratory of Quantitative Synthetic Biology Shenzhen Institute of Synthetic Biology Shenzhen Institutes of Advanced Technology Chinese Academy of Sciences Shenzhen China; ^3^ University of Chinese Academy of Sciences Beijing China; ^4^ Faculty of Synthetic Biology Shenzhen University of Advanced Technology Shenzhen China; ^5^ Department of Scientific Research and Teaching Shenzhen People's Hospital the First Affiliated Hospital Southern University of Science and Technology Shenzhen China; ^6^ Shenzhen Bay Laboratory Shenzhen China; ^7^ State Key Laboratory of Natural and Biomimetic Drugs School of Pharmaceutical Sciences Peking University Beijing China

**Keywords:** C‐end degron, influenza virus, live attenuated vaccine, proteolysis‐targeting vaccine, the ubiquitin‐proteasome system

## Abstract

Harnessing the cell's ubiquitin‐proteasome system (UPS) to manipulate viral protein degradation represents a promising way to develop live attenuated vaccines. We previously developed a proteolysis‐targeting (PROTAR) vaccine technology by artificially fusing a proteasome‐targeting degron (PTD) to the C terminus of influenza A viral M1 protein. Given the requirement of the PROTAR vaccine technology for PTD to be placed at the C terminus of viral proteins, we assumed that this technology could be generalized to the naturally occurring C‐end degrons. To explore this, we generated PROTAR vaccine strains by individually incorporating three C‐end degrons at the C terminus of influenza viral M1 protein. All generated PROTAR vaccine strains, namely M1^C−degron−1^, M1^C−degron−2^, and M1^C−degron−3^, exhibited proteasome‐dependent viral M1 protein degradation and robust attenuation in conventional host cells, while maintaining efficient replication in engineered TEVp‐expressing cells suitable for large‐scale manufacturing. These vaccine strains also showed sufficient attenuation and safety in vivo. A single intranasal dose elicited potent humoral, mucosal, and markedly enhanced T cell immune responses, supported by PTD‐mediated increases in M1 antigen presentation. Importantly, the vaccines conferred strong protection against both homologous H1N1 and heterologous H3N2 infection, with heterologous immunity shown to be CD8^+^ T cell‐dependent rather than antibody‐mediated. This study demonstrates the general applicability of natural C‐end degrons in PROTAR vaccine design, expanding the scope and versatility of PROTAR live attenuated vaccine technology.

## Graphical abstract

Naturally occurring C‐end degrons are exploited to design proteolysis‐targeting live attenuated influenza vaccines. C‐end degron tagging of viral M1 protein promotes proteasome‐dependent degradation, resulting in robust attenuation in host cells while permitting scalable production in engineered cells. A single intranasal vaccination elicits strong mucosal, humoral, and CD8⁺ T cell responses, conferring broad protection against homologous and heterologous influenza viruses.

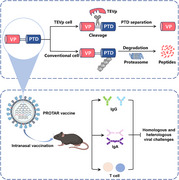

## 1 Introduction

Vaccination has been regarded as one of the most effective and cost‐efficient strategies for preventing and controlling infectious diseases such as influenza [1, 2, 3]. However, despite the availability of licensed influenza vaccines, influenza remains a significant global health threat [4]. The World Health Organization (WHO) estimates that influenza infects nearly 1 billion people annually, causing approximately 5 million cases of severe respiratory illness and up to 700 000 deaths worldwide [5, 6, 7]. These persistent public health and socioeconomic burdens underscore the need for new vaccine approaches that can be developed in a safer, more effective, and more straightforward manner [8].

We recently developed a proteolysis‐targeting (PROTAR) vaccine technology by harnessing the cell's ubiquitin‐proteasome system (UPS) to manipulate viral protein degradation for attenuation [9, 10]. In this design, a conditionally removable proteasome‐targeting degron (PTD) was artificially fused to the C terminus of the influenza A viral M1 protein via a tobacco etch virus cleavage site (TEVcs) linker. This design enabled PTD‐mediated M1 degradation and viral attenuation in conventional cells, while allowing PTD removal through TEVcs cleavage by TEV protease (TEVp) for vaccine manufacturing in TEVp‐expressing cells [9, 10]. A key characteristic of this PROTAR design is that the PTD must be positioned at the C terminus of the viral protein, as its removal is essential for efficient vaccine production. This requirement prompted us to hypothesize that naturally occurring C‐end degrons could serve as broadly applicable PTDs for PROTAR vaccine construction. C‐end degrons are short peptide motifs located at the extreme C terminus of eukaryotic substrate proteins [11, 12, 13]. They govern the degradation of a large subset of eukaryotic proteins by recruiting specific E3 ubiquitin ligases to mediate polyubiquitination and subsequent proteasomal degradation [12, 13]. Their defined position and established role in targeted protein turnover make C‐end degrons attractive candidates for constructing next‐generation PROTAR vaccines.

**FIGURE 1:**
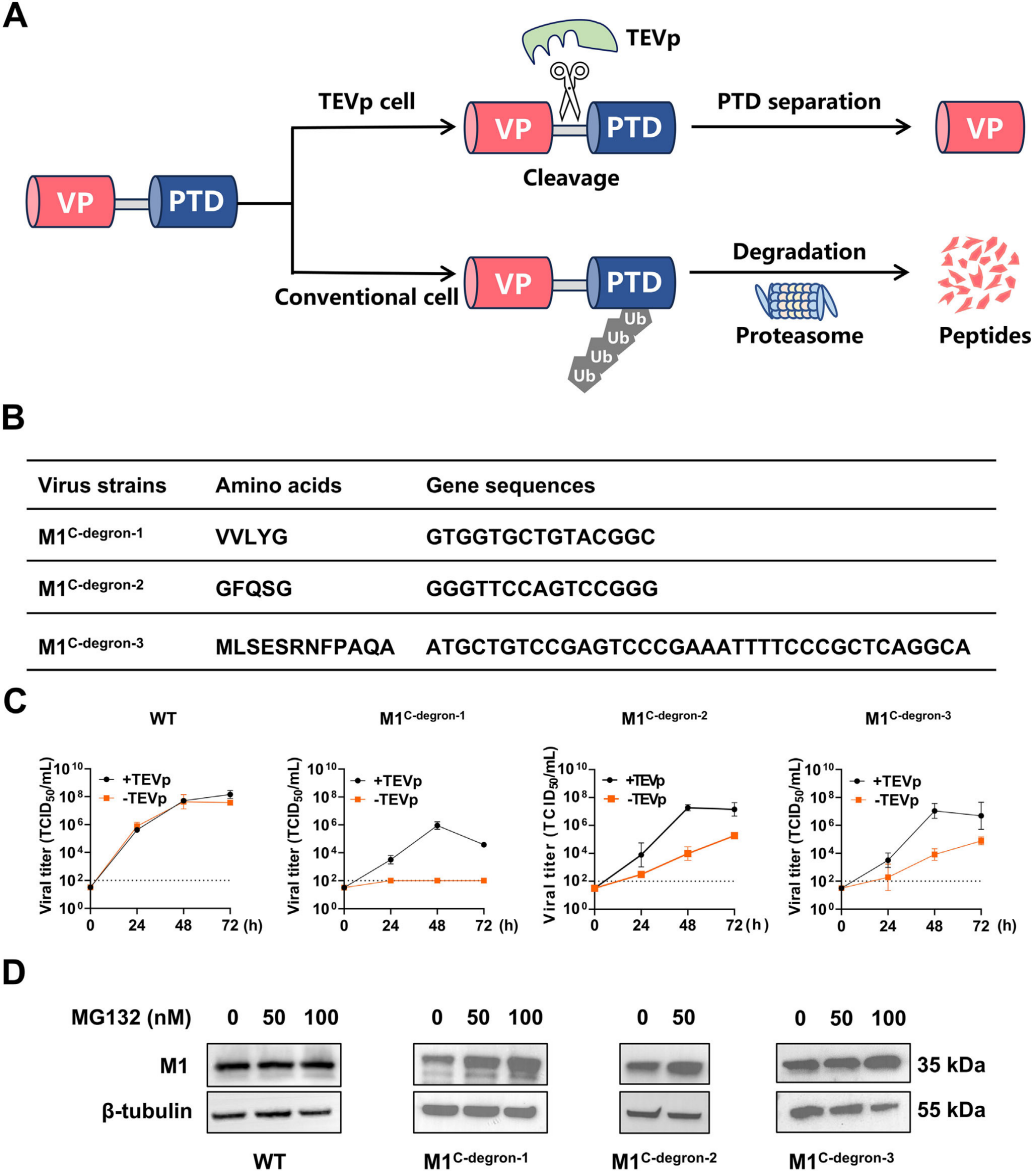
Generation of PROTAR viruses using C‐end degrons. A, Schematic diagram illustrating the principle of generation and attenuation of PROTAR viruses. B, Amino acid sequences and gene sequences of the C‐end degrons used in the study. C, Growth curves of WT, M1^C−degron−1^, M1^C−degron−2^, and M1^C−degron−3^ viruses in MDCK‐TEVp (+TEVp) and MDCK (‐TEVp) cells (n = 3). Data are expressed as mean ± s.d. The dashed line indicates the detection limit (10^2^ TCID_50_/mL). D, Western blots showing proteasome‐dependent degradation of M1 protein of M1^C−degron−1^, M1^C−degron−2^, M1^C−degron−3^ but not WT virus. MDCK cells were infected with the indicated viruses (MOI = 0.01) and cultured for 48 h in the presence or absence of MG‐132, followed by detection of viral M1 protein by western blotting (n = 3).

To explore the feasibility and general applicability of naturally occurring C‐end degrons in the development of PROTAR vaccines, we generated new PROTAR vaccine strains by individually incorporating three C‐end degrons at the C terminus of influenza viral M1 protein. All resulting M1^C−degron^ vaccine strains exhibited proteasome‐dependent M1 degradation and potent attenuation in conventional host cells, while maintaining efficient replication in TEVp‐expressing producer cells (Figure 1A). In animal studies, these M1^C−degron^ vaccine strains showed robust attenuation and safety, elicited strong humoral, respiratory mucosal, and T‐cell immune responses, and provided strong protection against challenges of both homologous H1N1 virus and heterologous H3N2 virus. Mechanistically, the cross‐reactive protection of M1^C−degron^ vaccines was mainly mediated by CD8^+^ T cell responses.

## 2 Results

### 2.1 Generation of M1^C−degron−1^, M1^C−degron−2^, and M1^C−degron−3^ Viruses

In our design, three C‐end degrons (VVLYG [13], GFQSG [13], and MLSESRNFPAQA [11]) were used as PTDs and individually fused to the C terminus of M1 protein of influenza A/WSN/33 (H1N1) virus via a TEVcs linker (ENLYFQG) (Figure 1B). In conventional cells, the PTDs function as proteasome‐targeting motifs that direct M1 for degradation. In contrast, in TEVp‐expressing cells, TEVp cleaves the TEVcs linker to remove the PTD from M1, thereby preventing its degradation and enabling efficient production of vaccine candidates (Figure 1A). Each engineered M1 construct was co‐transfected with the remaining plasmids of the influenza reverse genetics system into co‐cultures of TEVp‐expressing HEK293T (HEK293T‐TEVp) and MDCK (MDCK‐TEVp) cells, and viral supernatants collected at day 3‐4 were inoculated in MDCK‐TEVp cells, yielding three potential PROTAR vaccine strains: M1^C−degron−1^, M1^C−degron−2^, and M1^C−degron−3^ (Figure 1B).

We next measured the replication curves of M1^C−degron−1^, M1^C−degron−2^, M1^C−degron−3^, and wild‐type (WT) viruses in MDCK‐TEVp cells and conventional MDCK cells by infecting them (MOI = 0.001) and quantifying progeny virus titers at 24, 48, and 72 h using the Median Tissue Culture Infectious Dose (TCID_50_) assay. WT virus exhibited robust replication in both cell types, reaching ∼10^8^ TCID_50_/mL by 72 h (Figure 1C). In contrast, M1^C−degron−1^, M1^C−degron−2^, and M1^C−degron−3^ viruses replicated efficiently in MDCK‐TEVp cells but were severely attenuated in conventional MDCK cells. By 48–72 h post‐infection, viral titers of M1^C−degron−1^, M1^C−degron−2^, and M1^C−degron−3^ in MDCK cells were approximately 10^2^, 10^5.3^, and 10^4.9^ TCID_50_/mL, representing 10^4.0^, 10^2.0^, and 10^2.1^‐fold reductions compared with their titers in MDCK‐TEVp cells (Figure 1C). These data suggest that the replication of M1^C−degron−1^, M1^C−degron−2^, and M1^C−degron−3^ viruses is dependent on TEVp‐mediated removal of the PTD tag, resulting in strong attenuation in conventional host cells.

We next verified that the M1 protein degradation and attenuation phenotypes of M1^C−degron−1^, M1^C−degron−2^, and M1^C−degron−3^ viruses are proteasome‐dependent. MDCK cells were infected with M1^C−degron−1^, M1^C−degron−2^, M1^C−degron−3^, or WT virus and cultured for 48 h in the presence or absence of the proteasome inhibitor MG‐132. Western blot analysis revealed that proteasome inhibition had no effect on M1 protein levels in WT virus‐infected cells, whereas it substantially restored M1 protein levels in all three M1^C−degron^ viruses (Figure 1D). These findings demonstrate that the degron‐induced attenuation of the vaccine strains results from proteasomal degradation of M1 in conventional host cells. Together with the TEVp‐dependent rescue of replication (Figure 1C), these results confirm that incorporation of C‐end degrons enables precise control of viral replication: robust growth in TEVp‐expressing production cells and strong attenuation via M1 degradation in normal host cells. To assess genetic stability, all three M1^C−degron^ vaccine strains were serially passaged ten times in MDCK‐TEVp cells and subjected to genome sequencing. No mutations were detected in any of the C‐end degron sequences (Figure S1), confirming that the C‐end degron modifications remain genetically stable during multi‐round replication.

### 2.2 M1^C−degron−1^, M1^C−degron−2^, and M1^C−degron−3^ Viruses are Highly Attenuated in Vivo

**FIGURE 2:**
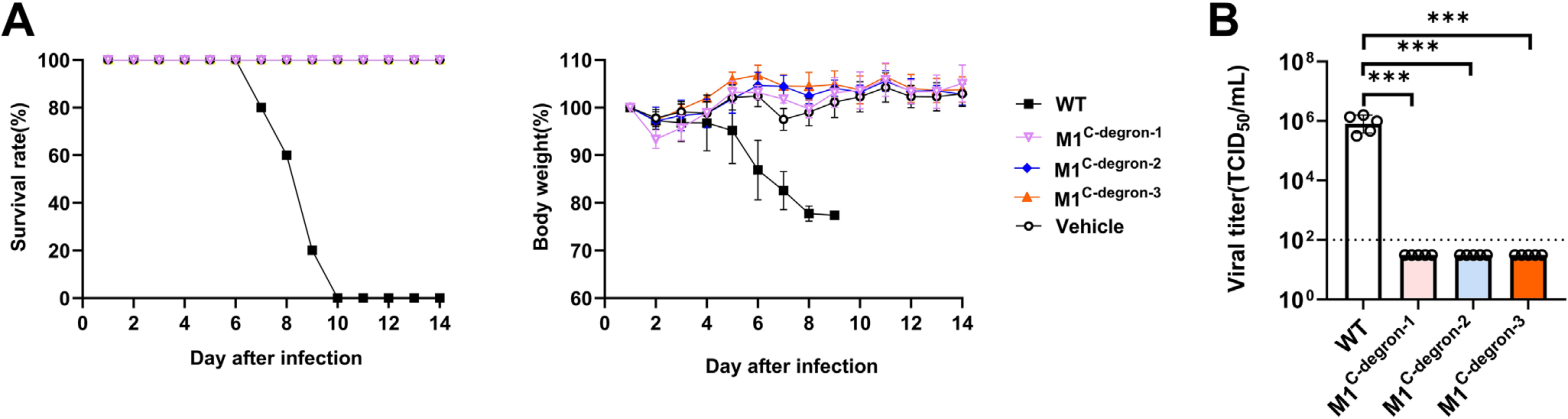
Evaluation of the in vivo safety of M1^C−degron−1^, M1^C−degron−2^, and M1^C−degron−3^ viruses in C57BL/6J mice. A, Survival rates (left) and body weight changes (right) of C57BL/6J mice (n = 5) after intranasal infection with 10^5^ TCID_50_ of WT, M1^C−degron−1^, M1^C−degron−2^, or M1^C−degron−3^ viruses. Data are expressed as mean ± s.d. B, Viral titers in lung tissues of mice (n = 5) on day 3 after intranasal infection with 10^5^ TCID_50_ of WT, M1^C−degron−1^, M1^C−degron−2^, or M1^C−degron−3^ viruses. Data are expressed as mean ± s.d.; one‐way ANOVA with Dunnett's multiple‐comparison test; ***, *P* < 0.001.

We next evaluated the in vivo attenuation of M1^C−degron−1^, M1^C−degron−2^, and M1^C−degron−3^ viruses in C57BL/6J mice. Animals were intranasally inoculated with 10^5^ TCID_50_ of WT WSN virus or the same dose of M1^C−degron−1^, M1^C−degron−2^, or M1^C−degron−3^ virus and monitored for body weight and survival over 14 days. WT WSN infection caused rapid and progressive weight loss, with all mice succumbing by day 10 (Figure 2A). In contrast, all mice infected with any of the three M1^C−degron^ viruses remained healthy, maintained stable body weight, and exhibited no clinical signs throughout the observation period (Figure 2A). To quantify viral replication in vivo, lung tissues were collected on day 3 post‐infection for TCID_5_₀ analysis. WT WSN virus replicated to ∼10^6^ TCID_5_₀/mL, whereas all three M1^C−degron^ viruses were undetectable at the assay limit (10^2^ TCID_5_₀/mL), indicating >10^4^‐fold attenuation relative to WT WSN virus (Figure 2B). These data suggest that incorporation of C‐end degrons enables strong attenuation of influenza A virus in vivo, yielding the M1^C−degron−1^, M1^C−degron−2^ and M1^C−degron−3^ strains as highly safe vaccine candidates.

Given the importance of the potential risk of gene reassortment with circulating influenza viruses, we performed in vitro and in vivo co‐infection of WT WSN virus and M1^C−degron^ viruses and detected virulence of progeny viruses. In the in vitro assay, MDCK cells were infected with WT WSN virus alone, or co‐infected with WT virus and each of the three M1^C−degron^ viruses. Notably, co‐infection resulted in markedly reduced progeny viral titers compared with WT virus‐only infection (Figure S2A), indicating that co‐infection did not generate more virulent progeny viruses, but instead the presence of M1^C−degron^ viruses reduced replication fitness of progeny viruses. This is potentially due to that reassortment of gene segments between M1^C−degron^ and WT viruses could lead to progeny viruses acquiring degron‐containing M1 gene segment, thereby impairing overall replication fitness. This attenuation effect was further confirmed in vivo. In C57BL/6J mice, co‐infection with WT WSN and M1^C−degron−3^ viruses resulted in significantly improved survival, reduced weight loss, and lower titers of progeny viruses in lungs compared with infection with WT WSN virus alone (Figure S2B,C). These findings demonstrate that co‐infection of M1^C−degron^ and WT viruses did not increase but reduce virulence of progeny viruses, alleviating safety concerns caused by co‐infection of M1^C−degron^ with circulating influenza viruses.

### 2.3 M1^C−degron−1^, M1^C−degron−2^, and M1^C−degron−3^ Vaccines Induce Strong and Broad Immune Responses

**FIGURE 3:**
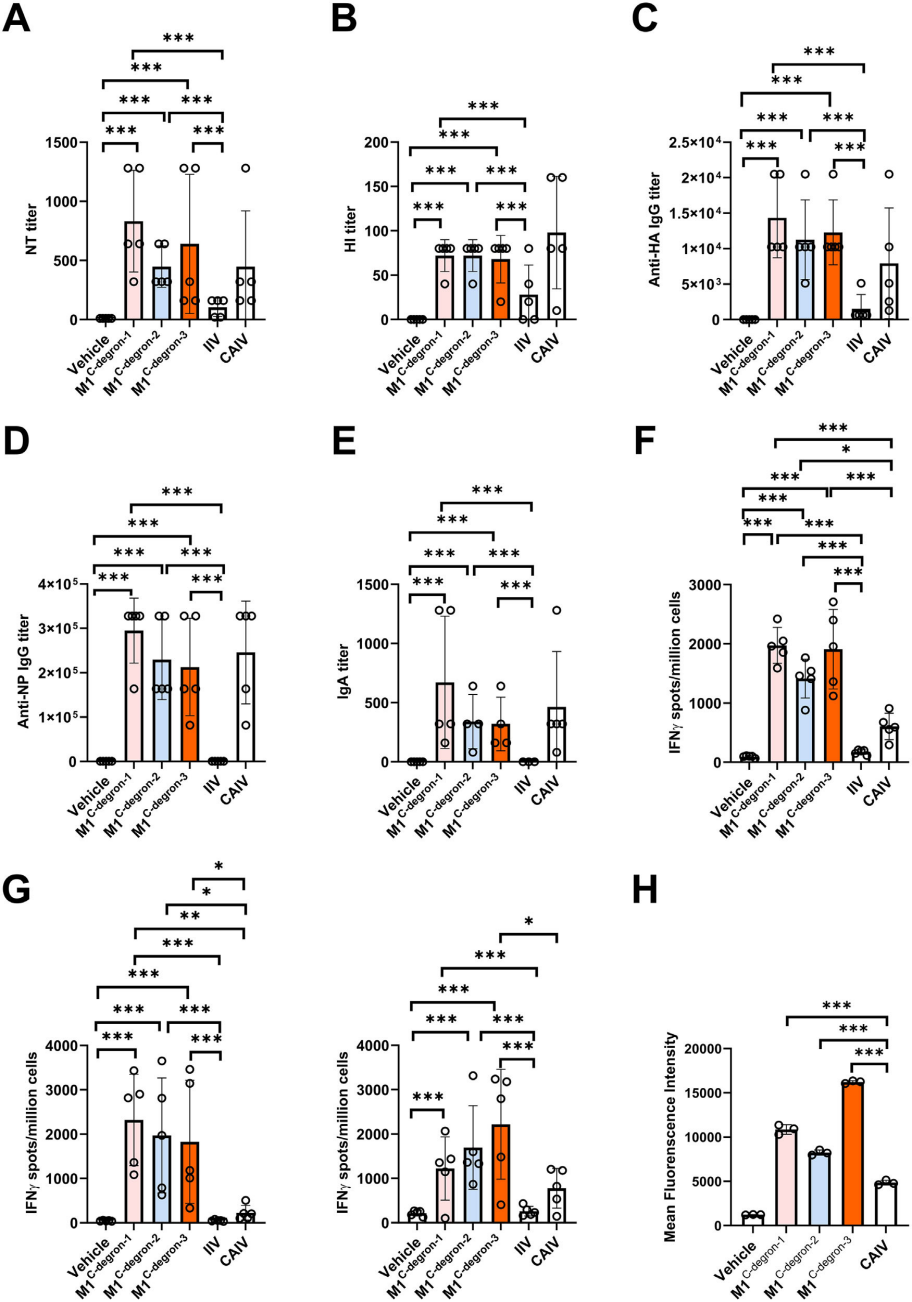
Evaluation of the immunogenicity of M1^C−degron−1^, M1^C−degron−2^, and M1^C−degron−3^ vaccines in C57BL/6J mice. A‐D, Serum antibody responses on day 21 after vaccination with 10^5^ TCID_50_ of the indicated vaccines (n = 5): NT (A), HI (B), anti‐HA IgG (C), and anti‐NP IgG (D). E, Virus‐specific IgA antibody responses in mouse lungs on day 21 after vaccination with 10^5^ TCID_5_₀ of the indicated vaccines (n = 5). F, Viral M1‐specific T cell responses in mouse lungs on day 7 after vaccination with 10^5^ TCID_5_₀ of the indicated vaccines (n = 5). IFN‐γ‐expressing cells per million cells are shown. G, Viral NP‐specific T cell responses in mouse lungs (left) and spleens (right) on day 7 after vaccination with 10^5^ TCID_5_₀ of the indicated vaccines (n = 5). IFN‐γ‐expressing cells per million cells are shown. H, PTD‐mediated enhancement of M1 antigen presentation in Raw264.7 cells. Cells were infected with M1^C−degron^ viruses (MOI = 1), and surface presentation of the M1_128‐135_ epitope was detected 6 h post‐infection using an anti‐M1 peptide (M1_128‐135_; MGLIYNRM) antibody. Data are expressed as mean ± s.d.; one‐way ANOVA with Tukey's multiple‐comparisons test. *, *P* < 0.05; **, *P* < 0.01; ***, *P* < 0.001.

We next evaluated the immunogenicity of the M1^C−degron−1^, M1^C−degron−2^, and M1^C−degron−3^ vaccine strains in C57BL/6J mice, using the most widely used inactivated influenza vaccine (IIV) and a classical cold‐adapted live attenuated influenza vaccine (CAIV) as controls. Mice were intranasally vaccinated with 10^5^ TCID_50_ of M1^C−degron−1^, M1^C−degron−2^, M1^C−degron−3^ or CAIV, or intramuscularly injected with an equivalent dose of IIV. One week after vaccination, mouse lungs and spleens were collected for assessment of antigen‐specific T cell responses by an interferon‐γ enzyme‐linked immunospot (IFN‐γ ELISpot) assay. Three weeks after vaccination, mouse serum and bronchoalveolar lavage (BAL) fluid were collected for assessment of systemic and mucosal antibody responses by neutralization (NT) assay, hemagglutination inhibition (HI) assay, and enzyme‐linked immunosorbent assay (ELISA). M1^C−degron−1^, M1^C−degron−2^, and M1^C−degron−3^ vaccines elicited high titers of NT antibodies, HI antibodies, viral surface HA protein‐specific immunoglobulin G (IgG) antibodies, and viral internal NP protein‐specific IgG antibodies in mouse serum (Figure 3A–D). The levels of these antibody responses were 2‐ to 460‐fold higher than those elicited by the IIV (Figure 3A–D). In addition, M1^C−degron−1^, M1^C−degron−2^, and M1^C−degron−3^ vaccines but not the IIV elicited antigen‐specific respiratory mucosal IgA antibody immune responses in mouse lungs (Figure 3E). All three M1^C−degron^ vaccines also generated markedly stronger M1‐ or NP‐specific T‐cell responses than CAIV in mouse lungs and spleens. Lung‐resident IFN‐γ^+^ T‐cell frequencies increased by 2‐ to 11‐fold, and splenic responses by 1.6‐ to 3‐fold relative to CAIV, whereas IIV induced only background‐level responses (Figure 3F,G). Mechanistically, this enhanced T‐cell immunity could be associated with increased antigen presentation: 6 h after infection, Raw264.7 macrophages infected with M1^C−degron−1^, M1^C−degron−2^, or M1^C−degron−3^ exhibited substantially elevated surface presentation of the M1_128‐135_ epitope compared with CAIV (Figure 3H). These data suggest that M1^C−degron−1^, M1^C−degron−2^, and M1^C−degron−3^ vaccines elicit strong and broad immunity, including humoral, respiratory mucosal, and T cell immune responses.

### 2.4 M1^C−degron−1^, M1^C−degron−2^, and M1^C−degron−3^ Vaccines Provide Cross‐Reactive Protection

**FIGURE 4:**
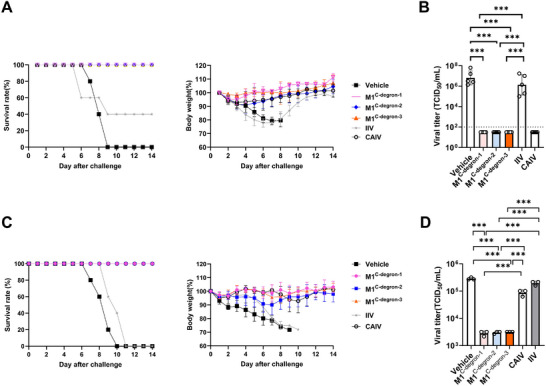
Evaluation of the protective efficacy of M1^C−degron−1^, M1^C−degron−2^, and M1^C−degron−3^ vaccines against homologous and heterologous virus in C57BL/6J mice. A, Survival rates (left) and body weight changes (right) of mice vaccinated with 10^5^ TCID_50_ of the indicated vaccines after intranasal challenge with 10^5^ TCID_50_ of WT WSN virus (n = 5). Data are expressed as mean ± s.d. B, Lung viral titers on day 3 after challenge with 10^5^ TCID_50_ of WT‐WSN virus in mice vaccinated with 10^5^ TCID_50_ of the indicated vaccines (n = 5). C, Survival rates (left) and body weight changes (right) of mice vaccinated with 10^5^ TCID_50_ of the indicated vaccines after intranasal challenge with 5 × LD_50_ of A/HK/8/68 (H3N2) virus (n = 5). Data are expressed as mean ± s.d. The dashed line indicates the detection limit (10^2^ TCID_50_/mL). D, Lung viral titers on day 3 after challenge with 5 × LD_50_ of H3N2 virus in mice vaccinated with 10^5^ TCID_50_ of the indicated vaccines (n = 5). Data are expressed as mean ± s.d.; one‐way ANOVA with Tukey's multiple‐comparison test; ***, *P* < 0.001.

Next, we investigated the ability of M1^C−degron−1^, M1^C−degron−2^, and M1^C−degron−3^ vaccines to confer protection against both homologous and heterologous influenza virus infection in C57BL/6J mice. Mice were intranasally vaccinated with 10^5^ TCID_50_ of each M1^C−degron^ vaccine or CAIV, or intramuscularly injected with an equivalent dose of IIV. Three weeks later, mice were intranasally challenged with 10^5^ TCID_50_ of homologous WT WSN virus and monitored for the body weight, survival, and disease symptoms for 14 days. All unvaccinated animals and 60% of IIV‐vaccinated mice succumbed to WSN infection within 9 days and exhibited rapid weight loss (Figure 4A). In contrast, all mice vaccinated with M1^C−degron−1^, M1^C−degron−2^, or M1^C−degron−3^ survived the viral challenge without measurable weight loss or disease symptoms (Figure 4A). Consistently, lung viral titers on day 3 post‐challenge reached 10^6^–10^7^ TCID_5_₀/mL in unvaccinated or IIV‐vaccinated mice, whereas no detectable virus was recovered from M1^C−degron^‐vaccinated animals (Figure 4B).

To determine whether M1^C−degron^ vaccines confer protection against heterologous influenza virus infection, we next challenged vaccinated mice with a lethal dose of influenza A/Hong Kong/8/68 (HK/68, H3N2) virus. All M1^C−degron^‐vaccinated and CAIV‐vaccinated mice survived with no weight loss, whereas Vehicle‐ and IIV‐treated mice succumbed rapidly (Figure 4C). Viral titration of lung tissues on day 3 post‐challenge revealed that viral loads in M1^C−degron^‐vaccinated mice were approximately 100‐, 30‐, and 70‐fold lower than those in unvaccinated, CAIV‐vaccinated, or IIV‐vaccinated mice, respectively (Figure 4D). These results demonstrate that M1^C−degron^ vaccines provide robust protection not only against homologous H1N1 infection but also against a completely heterologous H3N2 infection. Importantly, the efficacies of M1^C−degron^ vaccines against heterologous H3N2 infection were stronger than CAIV (Figure 4D), which could be ascribed to the higher levels of T‐cell immunity induced by M1^C−degron^ vaccines over CAIV (Figure 3F,G).

**FIGURE 5:**
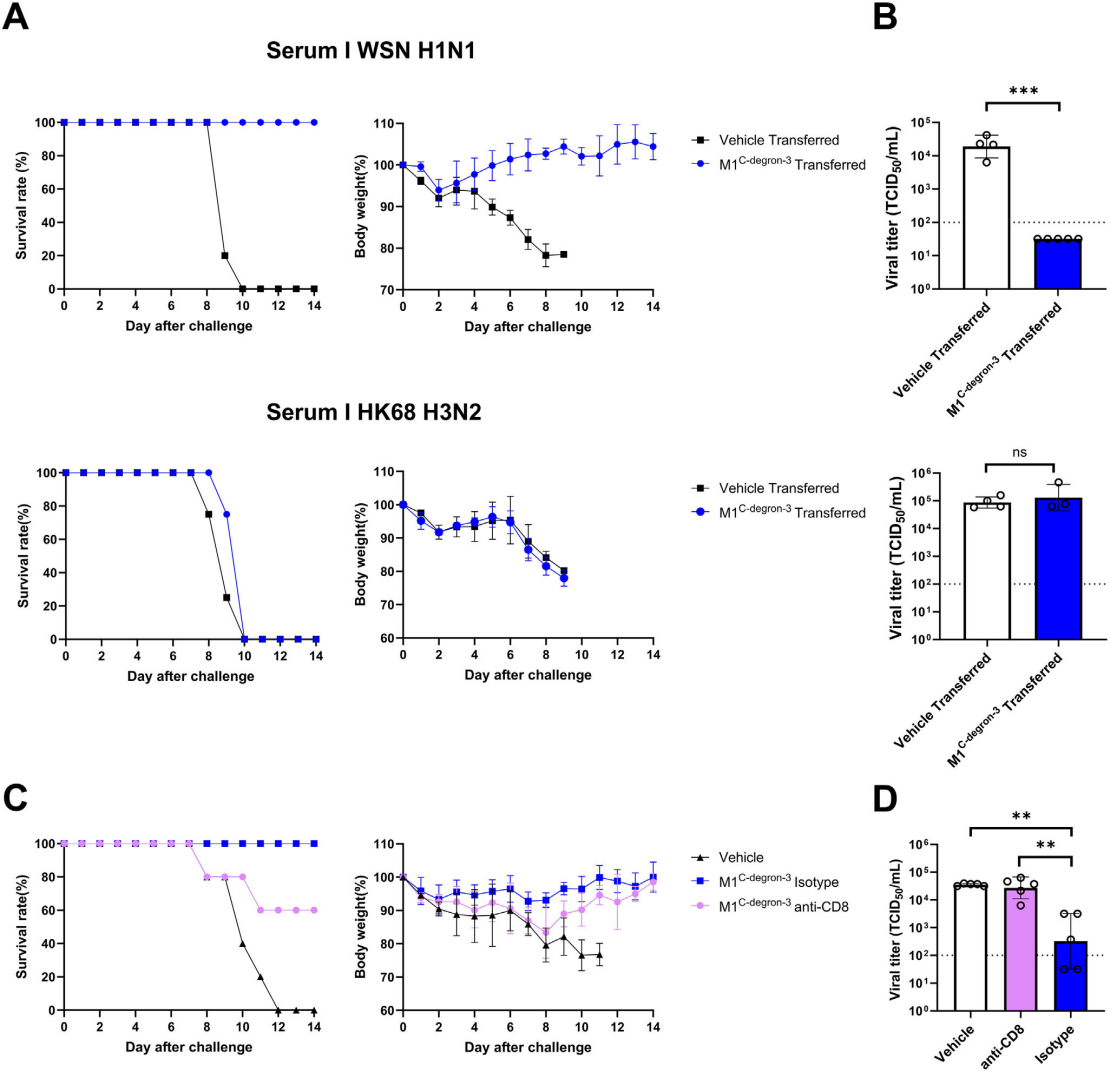
Passive serum‐transfer and CD8^+^ T‐cell depletion assays demonstrate the mechanisms of homologous and heterologous protection. A, Survival rates and body weight changes of naïve recipient mice that received serum from Vehicle controls or M1^C−degron−3^‐vaccinated donors. Mice were subsequently challenged with either 10^5^ TCID_50_ of WT WSN virus (upper panels) or 5 × LD_50_ of A/HK/8/68 (H3N2) virus (lower panels) (n = 5). Data are expressed as mean ± s.d. B, Lung viral titers on day 3 post‐challenge in the corresponding serum‐transfer groups for WSN (upper) and H3N2 (lower) challenges. The dashed line indicates the detection limit (10^2^ TCID_50_/mL). Data are expressed as mean ± s.d. Comparisons between two groups were analyzed using two‐tailed Student's *t*‐test. ns, not significant; ***, *P* < 0.001. C, Survival rates (left) and body weight changes (right) of M1^C−degron−3^‐vaccinated mice depleted of CD8^+^ T cells or treated with isotype control antibody prior to challenge with 10 × LD_50_ of A/HK/8/68 (H3N2) virus (n = 5). Data are expressed as mean ± s.d. D, Lung viral titers on day 3 post‐challenge in CD8^+^ T‐cell‐depleted or isotype control‐treated M1^C−degron−3^‐vaccinated mice, compared with Vehicle controls (n = 5). The dashed line indicates the detection limit (10^2^ TCID_50_/mL). Data are presented as mean ± s.d.; one‐way ANOVA with Tukey's multiple‐comparisons test; **, *P* < 0.01.

To determine whether heterologous protection was mediated by antibodies, we performed passive serum‐transfer experiments. Passive transfer of serum from M1^C−degron−3^‐vaccinated mice completely protected recipient mice against homologous WSN infection (Figure 5A,B, **Top**), confirming that the M1^C−degron−3^‐induced serum antibodies are functional and protective in an antigen‐matched context. In contrast, passive transfer of the same serum did not protect against heterologous H3N2 infection (Figure 5A,B, **Bottom**). Recipient mice exhibited progressive weight loss, death, and high levels of lung viral titers (Figure 5A,B, **Bottom**). Consistent with these findings, NT, HI, and ELISA assays revealed no detectable cross‐reactive antibody responses against HK/68 (H3N2) in serum of M1^C−degron−3^‐vaccinated mice (Figure S3). These results indicate that antibody responses elicited by M1^C−degron^ vaccination are insufficient to mediate cross‐reactive protection against H3N2.

We next examined the role of CD8^+^ T cells in mediating cross‐reactive protection by depleting CD8^+^ T cells prior to H3N2 challenge. Consistent with the results in Figure 4C,D, M1^C−degron−3^ vaccination conferred protection against H3N2 challenge (Isotype group) (Figure 5C,D). However, depletion of CD8^+^ T cells prior to H3N2 challenge substantially impaired the protective efficacy of M1^C−degron−3^: reducing survival from 100% to 60%, causing weight loss, and restoring viral titers in mouse lungs to the levels comparable to those in unvaccinated mice (Figure 5C,D). These results demonstrate that CD8^+^ T cell responses rather than antibody responses induced by the M1^C−degron^ vaccines mainly contribute to the cross‐reactive protection against H3N2.

## 3 Discussion

Recent years have witnessed the development of diverse live attenuated vaccine strategies with distinct design principles and features [9, 10, 14, 15, 16, 17, 18, 19, 20, 21, 22, 23, 24, 25, 26, 27, 28, 29, 30]. The central concept of PROTAR vaccine technology is to artificially transfer degrons from their native substrate proteins onto viral proteins of interest, thereby directing the degron‐tagged viral proteins toward proteasomal degradation and achieving viral attenuation. In our previous reports [9, 10], the PTDs artificially fused to the termini of influenza viral proteins via a TEVcs linker were derived from degrons located at various internal positions within their native substrates. A systematic screening of PTD incorporation sites revealed that only C‐terminal incorporation of PTDs into the influenza M1 protein supported successful generation of PROTAR influenza vaccines [9, 10]. Inspired by this, our present study focuses on exploring whether the naturally occurring C‐end degrons could be used as PTDs for the design of PROTAR influenza vaccines.

Naturally occurring C‐end degrons could provide several advantages over the previously used artificial PTDs in PROTAR vaccine design. First, naturally occurring C‐end degrons intrinsically function at the C terminus of native substrate proteins, they eliminate the need for empirical degron‐position screening and substantially simplify PROTAR vaccine design. In this study, all three C‐end degrons tested were successfully incorporated, whereas artificial PTDs previously required extensive trial‐and‐error optimization. Second, direct fusion of naturally occurring C‐end degrons without screening shortens the design‐to‐candidate timeline and reduces development cost, offering practical advantages for rapid vaccine deployment and pandemic preparedness. Third, naturally occurring C‐end degrons and internal degrons display distinct sequence and structural characteristics [31]. Internal degrons are often longer, more complex motifs whose activity sometimes requires conditional regulation, such as phosphorylation and hydroxylation. In contrast, naturally occurring C‐end degrons are generally short, linear, and simple peptide motifs. Therefore, utilizing C‐end degrons can not only minimize the impact of PTD incorporation on viral protein structure and function, but also streamline the factors to consider with PTD selection and PROTAR vaccine design.

Beyond the advantages over artificial PTDs, M1^C−degron^ vaccines also offer several important improvements relative to CAIV. First, M1^C−degron^ vaccines exhibit stronger attenuation than CAIV, with ∼10^2^‐ to 10^4^‐fold attenuation (Figure 1C) vs. ∼10^2^‐fold attenuation (Figure S4) at 37°C [18], providing an enhanced safety margin. Second, M1^C−degron^ vaccines demonstrate stronger cross‐reactive protection against heterologous H3N2 infection than CAIV in terms of viral titers in mouse lungs (Figure 4D). Third, M1^C−degron^ vaccines elicit higher levels of T‐cell immunity and enhanced viral antigen presentation through PTD‐mediated proteasomal degradation of M1 protein (Figure 3F–H), which is a unique feature of M1^C−degron^ vaccines compared with CAIV. Fourth, M1^C−degron^ vaccine approach can attenuate a circulating influenza virus strain into vaccine by fusing a C‐end degron to M1 protein of the virus, thereby providing complete antigen match between vaccine and target virus and thus potent immune efficacy. This is in contrast to CAIV that contains only HA and NA from circulating virus with all other components being from a cold‐adapted backbone virus. Fifth, M1^C−degron^ vaccines can be manufactured in mammalian cell systems, avoiding egg‐adaptation issues, allergy concerns, and scalability limitations associated with embryonated chicken egg‐based CAIV manufacturing. Finally, given a rich source of C‐end degrons has been identified recently [11, 13, 32], numerous C‐degron‐based PROTAR vaccine strains could be generated, which can increase the diversity of PROTAR vaccines and support systematic optimization of vaccine candidates.

Overall, this study establishes a new PROTAR vaccine strategy by leveraging naturally occurring C‐end degrons to attenuate virulent viruses into live vaccines, thereby broadening the scope and versatility of PROTAR vaccine technology. This approach could be broadly applicable for attenuating additional viral pathogens into live vaccines.

## 4 Materials and Methods

### 4.1 Viruses, Cells, and Vaccines

The influenza A/WSN/33 (H1N1) virus was generated in Madin‐Darby canine kidney (MDCK, ATCC, CRL‐2936) cells and Human embryonic kidney 293T (HEK293T, ATCC, CRL‐3216) cells using the 12‐plasmid rescue system in our laboratory. The influenza A/HK/8/68 (H3N2) virus was provided by Dr. M. Zheng from Shenzhen Bay Laboratory. HEK293T cells, MDCK cells, and Raw264.7 cells (Procell, CL‐0190) were maintained in DMEM (Gibco, C11995500BT) containing 10% fetal bovine serum (FBS, PAN, ST30‐3302), and 1% penicillin‐streptomycin (P/S, GENOM, GNM15140‐1) under standard culture conditions (37°C, 5% CO_2_). Stable TEVp‐expressing derivatives of MDCK (MDCK‐TEVp) and HEK293T (HEK293T‐TEVp) cells were previously generated in our laboratory [9].

### 4.2 Plasmids

The 12‐plasmid influenza A/WSN/33 (H1N1) virus rescue system [33] was kindly provided by Dr. George F. Gao from the Institute of Microbiology, Chinese Academy of Sciences. The plasmids for generating M1^C−degron−1^, M1^C−degron−2^, and M1^C−degron−3^ vaccine strains were constructed by inserting PTDs‐expressing genes (Figure 1B,C; Table S1) at the C terminal of M1 via a TEVcs linker (amino acids: GSGGENLYFQGGSG, gene sequence: GGTTCTGGTGGTGAGAATCTGTACTTCCAAGGTGGATCTGGA).

### 4.3 Virus Generation

HEK293T‐TEVp and MDCK‐TEVp cells were plated together in 6‐well plates (2 × 10^5^ and 5 × 10^4^ cells per well, respectively) and maintained for 24 h in DMEM containing 10% FBS for 24 h at 37°C in 5% CO_2_. Then the plasmids (0.2 µg/plasmid) for generating WT virus, M1^C−degron−1^, M1^C−degron−2^, or M1^C−degron−3^ vaccine strain were co‐transfected into the co‐cultured HEK293T‐TEVp and MDCK‐TEVp cells according to the instructions of TransIT‐X2 Dynamic Delivery System (Mirus, MIR6003). After 6 h, cultures were refreshed with infection medium (DMEM with 0.5% FBS, TPCK‐trypsin (2 µg/mL; Sigma‐Aldrich, T1426), and antibiotics) and maintained for 3–4 days. Supernatants were then transferred onto fresh MDCK‐TEVp cells to expand rescued viruses. When cytopathic effects (CPE) reached 80% ‐ 90%, the amplified viruses were collected and quantified using TCID_50_ assay.

Inactivated influenza vaccine (IIV) and cold‐adapted influenza vaccine (CAIV) were produced using the classical methods [18, 20, 34, 35, 36, 37]. Briefly, to generate IIV, WT A/WSN/33 virus was propagated in MDCK‐TEVp cells and inactivated by incubation with 0.2% formalin at 4°C for 7 days following classical IIV procedures. Complete inactivation was verified by inoculating formalin‐treated preparations onto MDCK cells and confirming absence of detectable infectivity by TCID_50_ assay. The resulting IIV preparation retains the HA and NA of the WSN backbone. A WSN‐based CAIV strain was generated using the 12‐plasmid reverse‐genetics system by introducing classical temperature‐sensitive and attenuating mutations into the internal genes of A/WSN/33, including PB2‐S265, PB1‐E391/E581/T661, and NP‐G34 [18]. HEK293T and MDCK cells were co‐transfected with plasmids encoding these mutated segments together with the remaining WSN gene segments. After transfection, cells were shifted to 33°C to rescue the cold‐adapted virus. Virus stocks were expanded in MDCK cells at 33°C, and cold‐adaption temperature‐sensitivity characterization was confirmed by incubating the virus at 33°C, 37°C, and 39°C in MDCK cells and determining viral titers using TCID_50_ assays (Figure S4) [18]. The CAIV preparation thus generated contains the HA and NA of the WSN backbone. Both IIV and CAIV were derived from the A/WSN/33 (H1N1) backbone and therefore express the same WSN‐matched HA used in all immunological assays (NT, HI, ELISA) and homologous challenge experiments, ensuring that all vaccine comparisons were performed under fully HA‐matched conditions.

### 4.4 TCID_50_ Assay

MDCK‐TEVp cells were plated in 96‐well plates at 5000 cells per well and cultured for 24 h at 37°C in 5% CO_2_, 100 µL of tenfold serially diluted viral samples in DMEM supplemented with 0.5% FBS, 2 µg/mL TPCK‐trypsin, and 1% P/S were added to each well. After another 4‐5 days of culture, CPE was recorded and used to calculate the viral titers using the Reed‐Muench method [38, 39].

### 4.5 Determination of Viral Growth Curves

MDCK and MDCK‐TEVp cells cultured in 6‐well plates (2 × 10^5^ cells/well) were infected with M1^C−degron−1^, M1^C−degron−2^, M1^C−degron−3^, or WT virus (MOI = 0.001) in DMEM supplemented with 0.5% FBS, 2 µg/mL TPCK‐trypsin, and 1% P/S. At 24, 48, and 72 h after infection, the culture supernatants were collected and subjected to TCID_50_ assay for viral quantification.

### 4.6 Analysis of Degradation of Viral M1 Protein by Proteasome

MDCK cells were seeded into 6‐well plates at 3 × 10^5^ cells per well and infected with M1^C−degron−1^, M1^C−degron−2^, M1^C−degron−3^, or WT virus (MOI = 0.01) in DMEM containing 0.5% FBS, 2 µg/mL TPCK‐trypsin, and 1% P/S for 2 h. Cultures were replaced with fresh infection medium (DMEM with 0.5% FBS, 2 µg/mL TPCK‐trypsin, and 1% P/S) supplemented with the indicated concentrations of MG‐132 (Sigma–Aldrich, M7449‐1mL). Cells were incubated for an additional 48 h at 37°C with 5% CO_2_, harvested, and lysed for western blot analysis of M1 levels. Primary antibodies included anti‐M1 (Sino Biological, 40010‐RP01, 1:1000 dilution) and anti‐β‐tubulin (Zen‐Bio, 700608, 1:10 000 dilution). Secondary antibody was HRP‐conjugated anti‐rabbit IgG (Proteintech, SA00001‐2, 1:2 000 dilution).

### 4.7 Detection of M1 Antigen Presentation by Flow Cytometry

M1 antigen presentation on the surface of Raw264.7 macrophages was assessed by flow cytometry. Raw264.7 cells were seeded into 12‐well plates at a density of 2 × 10^5^ cells per well in DMEM supplemented with 10% FBS and cultured for 24 h at 37°C in 5% CO_2_. The medium was then replaced with DMEM containing 1% FBS and 2 µg/mL TPCK‐treated trypsin, after which cells were infected with CAIV, M1^C−degron−1^, M1^C−degron−2^, or M1^C−degron−3^ viruses at an MOI of 1. At 6 h post‐infection, cells were harvested, washed with cold PBS, and incubated with an anti‐M1 monoclonal antibody (Sino Biological, 1:100 dilution), which recognizes the M1_128‐135_ epitope (MGLIYNRM). After washing, cells were stained with a CoraLite Plus 647‐conjugated goat anti‐rabbit secondary antibody (Proteintech, RGAR005, 1:500 dilution) for 30 min on ice in the dark. Stained cells were washed, resuspended in PBS containing 1% FBS, and analyzed on a Beckman Coulter flow cytometer. Data were processed using FlowJo software (version 10.8.1).

### 4.8 Mouse Studies

All animal experiments were conducted in strict accordance with the guidelines for the care and use of laboratory animals and were approved by the relevant institutional and national authorities. The animal experimental protocols were reviewed and approved by the Institutional Animal Care and Use Committee of the Shenzhen Institutes of Advanced Technology, Chinese Academy of Sciences (Approval No. SIAT‐IACUC‐240110‐HCS‐SLL‐A2450), the Institutional Animal Care and Use Committee of Sun Yat‐sen University (Shenzhen) (Approval No. 2024003169), the Ethics Committee for Animal Experiments of Shanxi Agricultural University (Approval No. SXAU‐EAW‐2025M.VZ.005063309), and the Institutional Review Board of the Brain Science Infrastructure of Shenzhen Institutes of Advanced Technology (Approval No. SIAT‐BSI‐IRB‐250821‐HCS‐SLL‐A0159). All procedures were performed in compliance with the approved protocols. 6‐8‐week‐old, female, specific‐pathogen‐free (SPF) C57BL/6J mice (Vital River Laboratory Animal Technology) were used for assessments of safety, immunogenicity, and protective efficacy of M1^C−degron−1^, M1^C−degron−2^, and M1^C−degron−3^ vaccine strains.

For evaluation of the in vivo safety of M1^C−degron−1^, M1^C−degron−2^, and M1^C−degron−3^ vaccine strains, ten mice per group were anesthetized with tribromoethanol and intranasally infected with 10^5^ TCID_50_ of WT virus, M1^C−degron−1^, M1^C−degron−2^, or M1^C−degron−3^. On day 3 post‐infection, five mice per group were euthanized for lung collection, homogenization in PBS, and viral titration by TCID_5_₀ assay. The remaining animals were monitored daily for body weight and survival for 14 days.

For evaluation of the immunogenicity of M1^C−degron−1^, M1^C−degron−2^, and M1^C−degron−3^ vaccine strains, groups of ten mice were anesthetized with tribromoethanol and intranasally inoculated with 10^5^ TCID_50_ of M1^C−degron−1^, M1^C−degron−2^, M1^C−degron−3^, or CAIV. An equivalent dose of IIV was administered intramuscularly, and DMEM served as the negative control (Vehicle). On day 7 post‐vaccination, five mice per group were euthanized, and lungs and spleens were collected for IFN‐γ ELISpot analysis of NP‐ and M1 peptide‐specific T‐cell responses. On day 21 post‐vaccination, serum and BAL fluid were collected from the remaining five mice for quantification of NT antibody, HI antibody, IgG antibody, and IgA antibody responses.

For evaluation of the protective efficacy of M1^C−degron−1^, M1^C−degron−2^, and M1^C−degron−3^ vaccine strains against homologous and heterologous influenza viruses, groups of ten mice were anesthetized with tribromoethanol and intranasally inoculated with 10^5^ TCID_50_ of M1^C−degron−1^, M1^C−degron−2^, M1^C−degron−3^, or CAIV, or intranasally inoculated with 10^5^ TCID_50_ of IIV. DMEM was used as the negative control (Vehicle). On day 21 after vaccination, mice were challenged with homologous influenza A/WSN/33 (H1N1) virus (10^5^ TCID_50_ per mouse) or heterologous influenza A/HK/8/68 (H3N2) virus (5 × LD_50_). On day 3 after the infection, five mice per group were euthanized, and lungs were collected, homogenized in PBS, followed by viral quantification using TCID_50_ assay. The remaining five mice were monitored daily for body weights and mortality for 14 days.

### 4.9 Passive Serum‐Transfer Experiment

Passive serum‐transfer studies were performed to assess the contribution of vaccine‐induced antibodies to homologous and heterologous protection. Mice were immunized with 10^5^ TCID_50_ of M1^C−degron^. Three weeks after immunization, blood was collected, and serum were isolated by centrifugation. All serum were heat‐inactivated at 56°C for 30 min prior to transfer. Recipient C57BL/6J mice received intraperitoneal injections of 200 µL of serum on days −4, −2, 0, and +1 of viral challenge. Following the third serum transfer, mice were challenged intranasally with either influenza A/WSN/33 (H1N1) virus (10^5^ TCID_50_) or A/HK/8/68 (H3N2) virus (5 × LD_50_) at the indicated doses. Mice were monitored daily for body weight and survival for 14 days post‐challenge.

### 4.10 CD8^+^ T Cell Depletion Experiment

CD8^+^ T cell depletion experiment was performed to assess the contribution of T cell‐mediated immunity to vaccine‐induced heterologous protection. Mice were immunized with 10^5^ TCID_50_ of M1^C−degron^ and allowed to rest for three weeks. CD8^+^ T cells were depleted by intraperitoneal injection of anti‐CD8α monoclonal antibody (clone 2.43, BioXCell) at 200 µg per dose on days −4, −2, 0, +2, and +4 of viral challenge. Control mice received an equivalent dosing schedule of isotype control antibody (A2116, Selleck). Lungs and spleens were collected one day prior to viral challenge to confirm efficient depletion (> 98%) by flow cytometry. Depleted and control mice were then challenged intranasally with A/HK/8/68 (H3N2) virus (10 × LD_50_). Mice were monitored daily for body weight and survival for 14 days following challenge.

### 4.11 NT Assay

The NT assay was performed as described previously [10, 14]. Mouse serum was pretreated with receptor‐destroying enzyme (RDE) (Denka Seiken, 340122) according to the manufacturer's instructions. MDCK cells were seeded into 96‐well plates and cultured to form monolayers. Serial dilutions of RDE‐treated sera were incubated with WT A/WSN/33 (H1N1) virus (MOI = 0.01) in DMEM supplemented with 1% FBS, 2 µg/mL TPCK‐trypsin, and 1% P/S. The virus‐serum mixtures were then added to MDCK monolayers and incubated for 3–5 days at 37°C in 5% CO_2_. CPE were assessed microscopically, and neutralizing antibody titers were defined as the highest serum dilution that completely prevented CPE.

### 4.12 HI Assay

The HI assay was performed as described previously [10, 14]. RDE‐treated serum was serially diluted in PBS and incubated with 4 HA units of WT virus for 30 min at room temperature. Chicken red blood cells (CRBCs) were resuspended in PBS to 1%. Then, 50 µL of the serum‐virus mixture was incubated with 50 µL of the chicken red blood cell solution in V‐shaped 96‐well plates. Following a 30‐min incubation at room temperature, hemagglutination patterns were recorded. HI titers were defined as the highest serum dilution that completely inhibited agglutination of CRBCs.

### 4.13 ELISA

The ELISA was performed to measure the titers of antigen‐specific IgG and IgA antibodies. 96‐well plates (Corning, 3590) were coated with 1 µg/mL of recombinant HA protein (Sino Biotech, 11692‐V08H), 1 µg/mL of recombinant NP protein (Sino Biotech, 11675‐V08B), or 10^6^ TCID_50_/mL of WT virus that was diluted in ELISA coating buffer (Solarbio, C1055) overnight at 4°C. The plates were washed three times with washing buffer (PBS containing 0.1% Tween 20, PBST) and incubated with 100 µL/well of blocking buffer (PBST containing 5% skim milk (YEASEN, 36120ES60)) for 1 h at 37°C. Serially diluted serum or BAL samples prepared in blocking buffer were added to the plates and incubated for 2 h at 37°C. After five washes with PBST, plates were incubated with HRP‐conjugated anti‐mouse IgG (Proteintech, SA00001‐1; 1:2000 dilution) or HRP‐conjugated anti‐mouse IgA (Abcam, ab97235; 1:2000 dilution) diluted in blocking buffer for 1 h at 37°C. Plates were washed three times with PBST and developed with TMB substrate (Beyotime, P0209) for 15 min, followed by addition of ELISA stop solution (Solarbio, C1058). Absorbance at 450 nm was measured using a BioTek Synergy H1 microplate reader.

### 4.14 IFN‐γ ELISpot

Influenza virus M1 peptide‐specific T cell responses were measured by IFN‐γ ELISpot assay (Mabtech, 3321‐4AST‐2) according to the instructions and references. Viral antigen peptides (M1_128‐135_: MGLIYNRM, NP_366–374_: ASNENMETME) were synthesized by GenScript and used as the stimuli.

### 4.15 In Vitro and in Vivo Co‐Infection of M1^C−degron^ Viruses with WT Virus

To test in vitro co‐infection, MDCK cells were seeded into 6‐well plates and cultured for 24 h at 37°C in 5% CO_2_. Cells were then infected with WT A/WSN/33 (H1N1) virus alone (MOI = 0.01), or co‐infected with WT virus (MOI = 0.01) together with each of the three M1^C−degron^ viruses (MOI = 0.1). After virus adsorption for 1 h, the inoculum was removed and replaced with DMEM containing 0.5% FBS, 2 µg/mL TPCK‐trypsin, and 1% P/S. Culture supernatants were collected at 24, 48, and 72 h post‐infection and clarified by centrifugation. Viral titers were quantified using a TCID_5_₀ assay on MDCK‐TEVp cells. Replication kinetics of progeny viruses were compared between WT‐only infection and WT + M1^C−degron^ co‐infection conditions.

To test in vivo co‐infection, groups of ten female C57BL/6J mice (6‐8 weeks old) were anesthetized with tribromoethanol and intranasally inoculated with DMEM (Vehicle control), 2 × 10^3^ TCID_5_₀ of WT WSN virus, or a mixture of 2 × 10^3^ TCID_5_₀ WT virus and 2 × 10^5^ TCID_5_₀ M1^C−degron^ virus. Five mice per group were euthanized on day 3 post‐infection, and lungs were harvested, homogenized in PBS, and titrated by TCID_5_₀ assay on MDCK‐TEVp cells. The remaining mice were monitored daily for body weight, clinical signs, and survival for 14 days. Differences in survival, body weight, and lung viral loads between WT‐only and WT + M1^C−degron^ groups were used to assess whether co‐infection altered virulence of progeny viruses.

### 4.16 Statistical Analysis

All experiments were independently performed at least three times. GraphPad Prism 9 was used for graphing and statistical analysis. Comparisons between the two groups were analyzed using two‐tailed Student's t‐test. Comparisons among three or more groups were analyzed using one‐way ANOVA and Tukey's or Dunnett's multiple comparison test. A *P* value < 0.05 was considered statistically significant. Data are expressed as mean ± s.d. Significance levels are indicated as follows: ns, not significant; *, *P* < 0.05;**, *P* < 0.01;***, *P* < 0.001.

## Author Contributions

L.S. conceived the study. P.W., L.L., and Y.C. conducted experiments and analyses, with other authors assisting in experiments and data analysis. L.S., L.T., P.W., L.L., and Q.Z. wrote the manuscript, and all authors provided feedback.

## Conflicts of Interest

The authors declare no conflicts of interest.

## Supporting information




**Supporting File**: advs74053‐sup‐0001‐SuppMat.docx

## Data Availability

The data that support the findings of this study are available from the corresponding author upon reasonable request.
