# Supplementary material for: Attenuation of Influenza a Virus into Live Vaccines Through C‐End Degrons
Source: Adv Sci (Weinh). 2026 Feb 21;13(19):e09425. doi: 10.1002/advs.202509425 (PMC13045484; doi:10.1002/advs.202509425)
Supplement: Supplementary file 1 — Supporting File: advs74053‐sup‐0001‐SuppMat.docx [file ADVS-13-e09425-s001.docx]

Supplementary Materials for

**Attenuation of influenza A virus into live vaccines**

**through C-end degrons**

Ping Wang^1,2,#^, Le Li^1,2,3,4,#^, Yunfang Chen^5,#^, Le Tong^2,#^, Zhen Li^2,6,#^, Rong Yu^1^, Quan Shen^2^, Qikai Wang^1,2^, Jihuan Hou^2^, Qisi Zhang^2^, Xu Si^2,3^, Ning Wang^2^, Demin Zhou^6,7^, Wen-xia Tian^1,*^, Longlong Si^2,3,1,*^

^1^Shanxi Key Laboratory of Animal Disease Research, Prevention and Control, College of Veterinary Medicine, Shanxi Agricultural University, Jinzhong 030801, China.

^2^State Key Laboratory of Quantitative Synthetic Biology, Shenzhen Institute of Synthetic Biology, Shenzhen Institutes of Advanced Technology, Chinese Academy of Sciences, Shenzhen 518055, China.

^3^University of Chinese Academy of Sciences, Beijing 100049, China.

^4^Faculty of Synthetic Biology, Shenzhen University of Advanced Technology, Shenzhen 518038, China.

^5^Department of Scientific Research and Teaching, Shenzhen People’s Hospital, the First Affiliated Hospital, Southern University of Science and Technology, Shenzhen, 518020, China.

^6^Shenzhen Bay Laboratory, Shenzhen, China.

^7^State Key Laboratory of Natural and Biomimetic Drugs, School of Pharmaceutical Sciences, Peking University, Beijing, China.

^#^These authors contributed equally to this work.

*Corresponding authors: wenxiatian@126.com; ll.si@siat.ac.cn

**M1^C-degron-1^**


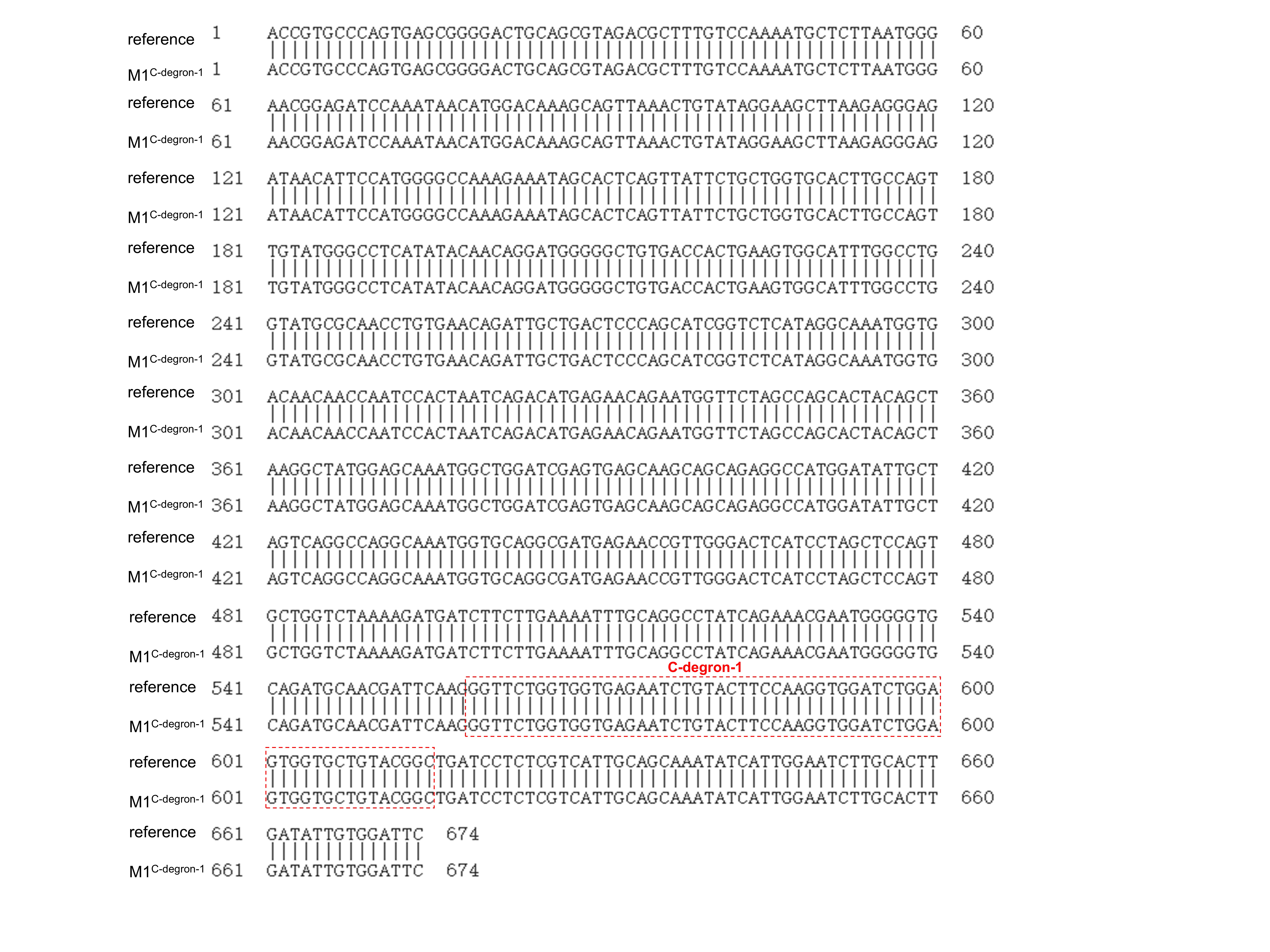


**M1^C-degron-2^**


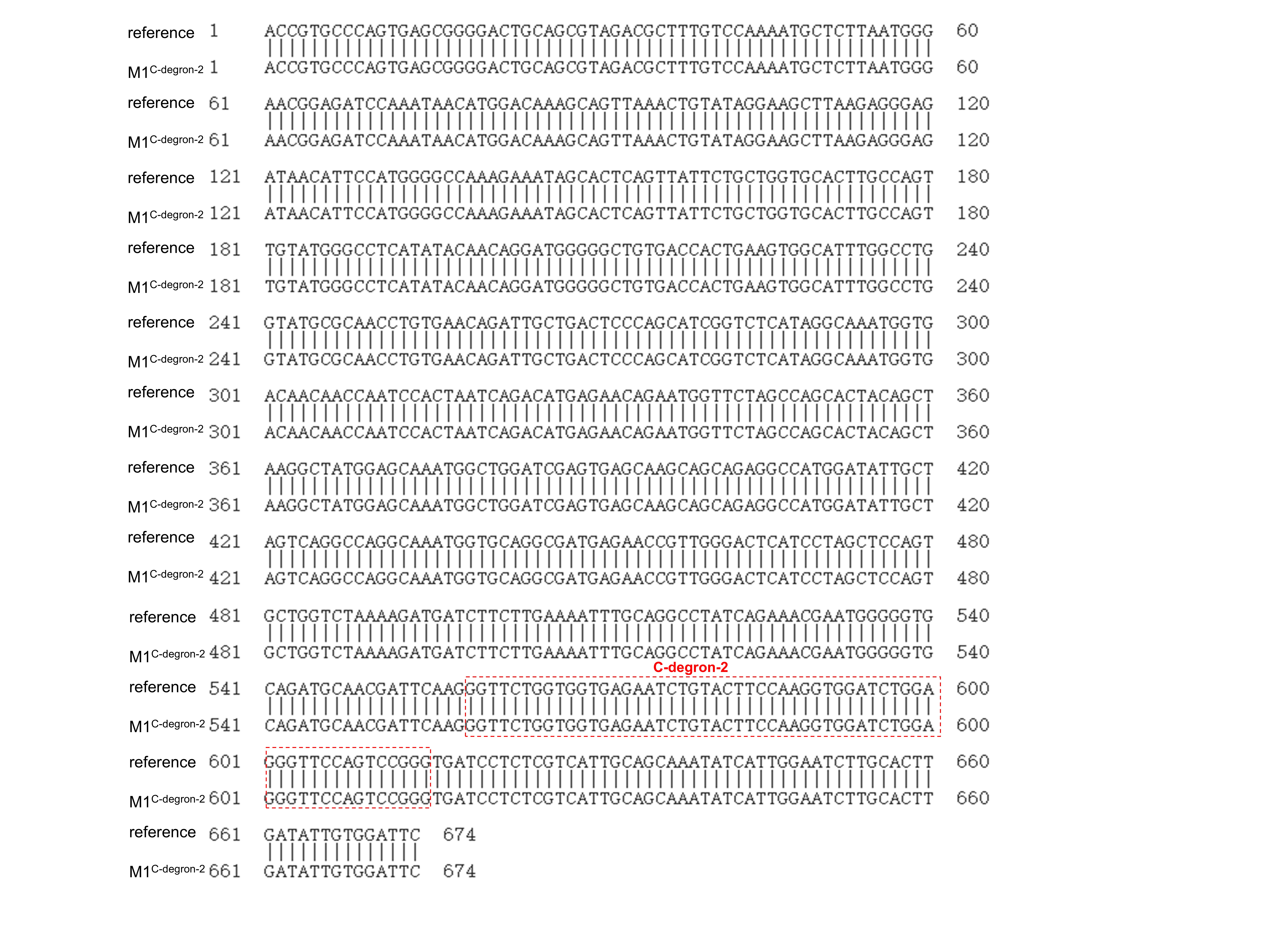


**M1^C-degron-3^**


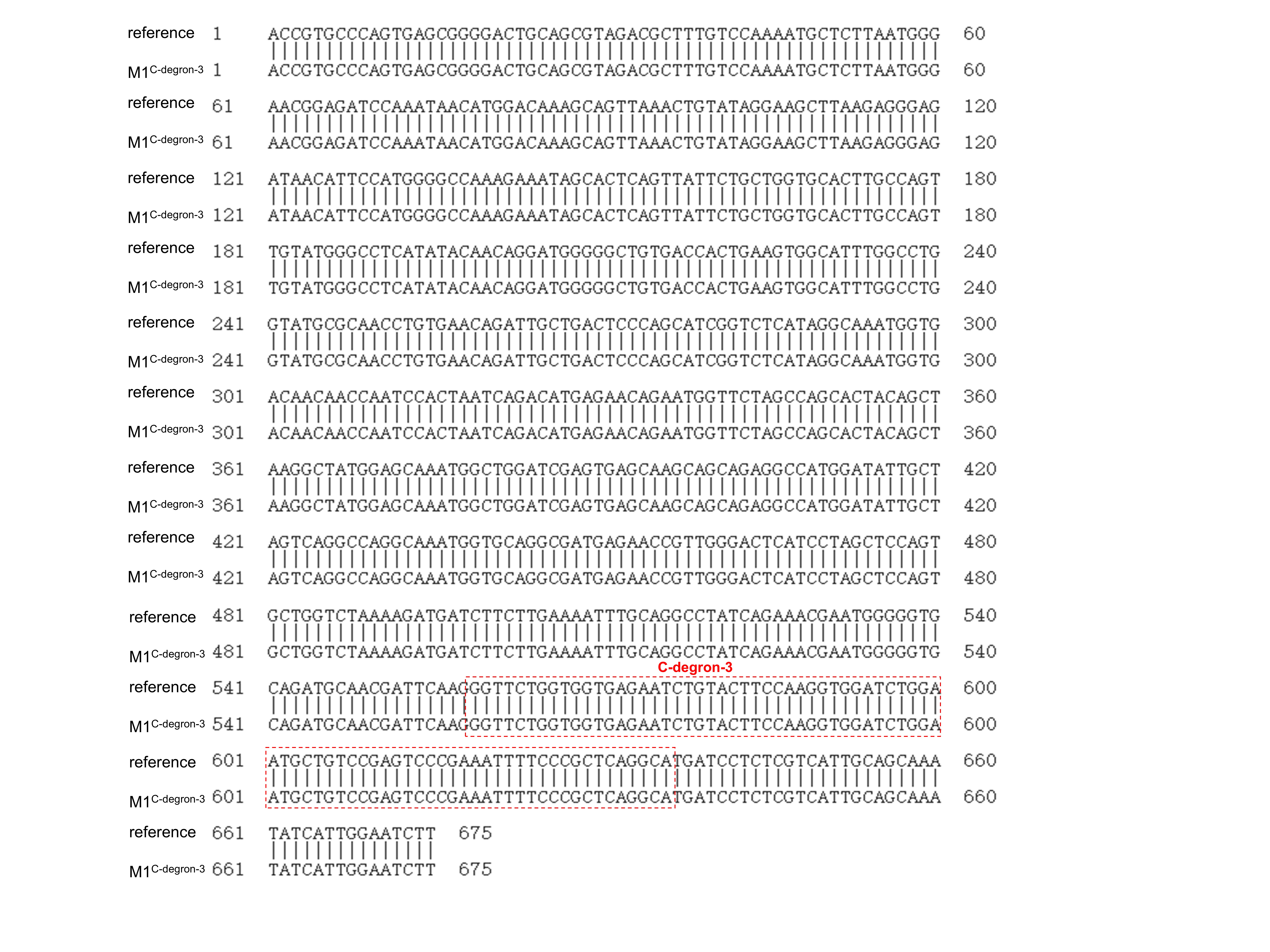


**Supplementary Fig. 1. Characterization of the genetic stability of** **M1^C-degron-1^, M1^C-degron-2^, and M1^C-degron-3^ viruses.** M1^C-degron^ viruses were serially passaged ten times in MDCK-TEVp cells and subjected to viral genome sequencing. Reference sequences corresponding to the engineered M1^C-degron-1^, M1^C-degron-2^, and M1^C-degron-3^ constructs were used for alignment. Sequencing analysis revealed no mutations within the C-end degron regions, demonstrating the genetic stability of the inserted degrons.





**Supplementary Fig. 2. Characterization of *in vitro* and *in vivo* co-infection of M1^C-degron^ viruses and WT virus.** **A**, MDCK cells were infected with WT WSN virus (MOI = 0.01) alone, or co-infected with WT virus (MOI = 0.01) and each of the three M1^C-degron^ viruses (MOI = 0.1). Supernatants were collected at 24, 48, and 72 h post-infection and titrated by TCID₅₀ assay. Progeny viruses exhibited reduced replication in all co-infection conditions compared with WT-only infection (n = 3). **B**, Survival rates (left) and body weight changes (right) of C57BL/6J mice intranasally inoculated with DMEM (Vehicle), 2 × 10^3^ TCID₅₀ of WT WSN virus, or a mixture of 2 × 10^3^ TCID_50_ of WT virus and 2 × 10^5^ TCID_50_ of M1^C-degron-3^ virus (n = 5). Co-infection with WT and M1^C-degron-3^ virus reduced morbidity and mortality with no evidence of increased virulence compared to WT virus-only infection. **C**, Lung viral titers measured on day 3 post-infection in the same experimental groups as in **B**. WT + M1^C-degron-3^ co-infection resulted in lower viral titers compared with WT alone (n = 5). Data are expressed as mean ± s.d.; one-way ANOVA with Tukey’s multiple-comparison test; ***, *P* < 0.001


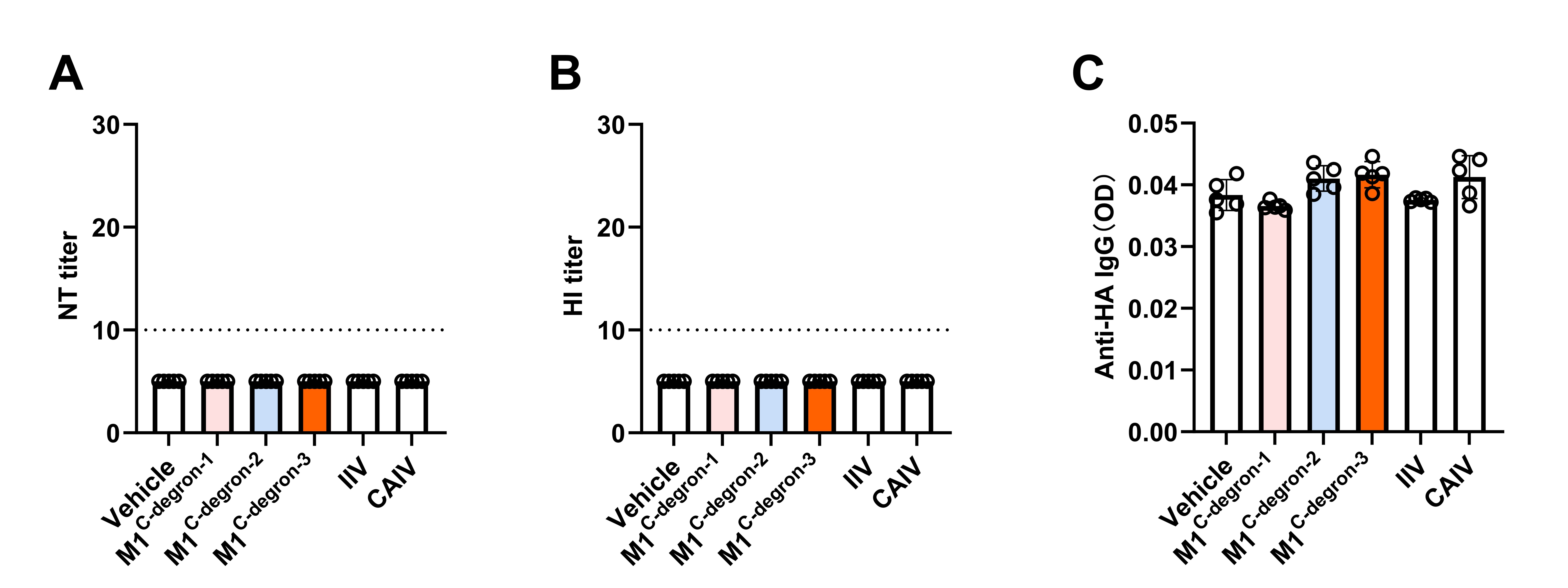


**Supplementary Fig. 3. Evaluation of heterologous A/HK/8/68 (H3N2)-specific antibody responses.** Serum collected on day 21 after vaccination with IIV, CAIV, M1^C-degron-1^, M1^C-degron-2^, or M1^C-degron-3^ vaccines were assessed for cross-reactive humoral responses against the heterologous A/HK/8/68 (H3N2) strain. **A**, NT titers against A/HK/8/68. **B**, HI titers against A/HK/8/68. **C**, A/HK/8/68 HA-specific IgG antibodies. Across all three assays, no cross-reactive antibody responses were detected in any vaccine group. Data are expressed as mean ± s.d. (n = 5).





**Supplementary Fig. 4.** **Cold-adapted and temperature-sensitive attenuation of CAIV**. Viral titers of influenza A/WSN/33 (H1N1) and CAIV were measured in MDCK-TEVp cells following incubation at 33 ℃, 37 ℃, and 39 ℃. CAIV exhibited efficient replication at 33 ℃ and attenuation at 37 ℃ and 39 ℃ compared with WT WSN virus, confirming its classical cold-adapted and temperature-sensitive phenotype. Data are expressed as mean ± s.d. (n = 3).

**Supplementary Table 1. Primers used in this study.**

| **Primer name** | **Primer sequence (5’-3’)** |
| --- | --- |
| M1^C-degron-1^-F | CTTCCAAGGTGGATCTGGAGTGGTGCTGTACGGCTGATCCTCTCGTCATT |
| M1^C-degron-1^-R | AATGACGAGAGGATCAGCCGTACAGCACCACTCCAGATCCACCTTGGAAG |
| M1^C-degron-2^-F | CTTCCAAGGTGGATCTGGAGGGTTCCAGTCCGGGTGATCCTCTCGTCATT |
| M1^C-degron-2^-R | AATGACGAGAGGATCACCCGGACTGGAACCCTCCAGATCCACCTTGGAAG |
| M1^C-degron-3^-F | GAGTCCCGAAATTTTCCCGCTCAGGCATGATCCTCTCGTCATTGCAGC |
| M1^C-degron-3^-R | CGGGAAAATTTCGGGACTCGGACAGCATTCCAGATCCACCTTGGAAGT |
